# The mediating role of sleep disturbance in the relationship between depression and cardiovascular disease

**DOI:** 10.3389/fpsyt.2024.1417179

**Published:** 2024-06-05

**Authors:** Feng Chen, Hao Lin, Yuansi Zhang, Yu Zhang, Linlin Chen

**Affiliations:** ^1^Department of Child Healthcare, Wenzhou People’s Hospital, Wenzhou, Zhejiang, China; ^2^Department of Gastroenterology, Pingyang Affiliated Hospital of Wenzhou Medical University, Wenzhou, Zhejiang, China; ^3^Department of Traditional Chinese Medicine, Wenzhou Yebo Proctology Hospital, Wenzhou, Zhejiang, China

**Keywords:** sleep disturbance, cardiovascular diseases, depression, mediating effect, logistic regression

## Abstract

**Background:**

Studies suggest that both depression and disrupted sleep disturbance are linked to cardiovascular disease (CVD). However, the precise role of sleep disturbance in the connection between depression and CVD is poorly understood. Therefore, we sought to examine the associations among these factors and further explore the mediating role of sleep disturbance in the association between depression and CVD.

**Methods:**

This study included data from 29,831 adults (≥20 years old). Multifactorial logistic regression analyses were conducted to examine the relationships among depression, sleep disturbance, and CVD. Additionally, bootstrap tests were used to investigate whether the association between depression and CVD was mediated by sleep disturbance.

**Results:**

Our research showed that individuals who experienced depression or sleep disturbance had a notably greater likelihood of developing CVD than those who did not have these issues (depression: OR: 2.21, 95% CI=1.96–2.49; sleep disturbance: OR: 1.74, 95% CI=1.6–1.9). Even after adjusting for potential confounders, depression was still positively associated with the risk of sleep disturbance (OR: 4.07, 95% CI=3.73–4.44). Furthermore, sleep disturbance significantly mediated the association between depression and CVD, with a mediating effect of 18.1%.

**Conclusion:**

Our study demonstrated that depression, sleep disturbance, and CVD are interrelated. The increased risk of CVD among patients with depression may be attributed to the mediating role of sleep disturbance. This finding underscores the importance of interventions focused on sleep disturbances as a means to address the connection between depression and CVD.

## 1 Introduction

Cardiovascular disease (CVD) and depression significantly contribute to the global health burden (1). Approximately 20% to 40% of individuals with heart disease exhibit symptoms of depression or meet the criteria for major depressive disorder (2). Likewise, the occurrence of CVD is more prevalent in those suffering from depression than in the general population (3). Increasing evidence supports a bidirectional relationship between depression and CVD, with numerous studies demonstrating a connection between depressive symptoms and increased risk of CVD (4–7). Furthermore, depression is closely associated with poorer outcomes in CVD patients (8–10), suggesting a bidirectional relationship where depression and CVD mutually exacerbate each other (11).

Addressing sleep disturbances within the framework of depression is crucial, given that most individuals with depression encounter various forms of sleep issues. Moreover, sleep disturbances are recognized as standalone risk factors for both the emergence and recurrence of depression, serving as critical indicators for the onset of depressive episodes (12, 13). Scientific assessments have revealed a reciprocal connection between disrupted sleep patterns and depressive symptoms (14). Previous studies have proposed that treating insomnia can significantly alleviate depression, and nearly all antidepressants affect sleep (15). Alterations in sleep patterns may also serve as indicators of the efficacy of treatments for depression (16).

Sleep disturbances not only exacerbate mental health conditions but are also pivotal in the development and progression of chronic physical health conditions. Research indicates that sleep disturbances such as insomnia and hypersomnia are intricately connected to the development of CVD and are associated with further complications such as intermittent hypoxia, oxidative stress, heightened sympathetic nervous system function, and endothelial dysfunction—key factors contributing to the onset and progression of CVD (17–20). For instance, the treatment of sleep apnoea, which is associated with the incidence and morbidity of CVD, has the potential to improve cardiovascular health by reducing oxidative stress and improving endothelial function (21–23). An analysis conducted systematically and through meta-analysis revealed that individuals exhibiting symptoms of insomnia face a 45% increased likelihood of developing CVD or succumbing to CVD in comparison to individuals who do not demonstrate signs of insomnia (17). Therefore, promptly addressing and effectively treating sleep disturbances is vital for both the prevention and control of CVD.

According to previous studies, there is a strong connection between sleep disturbances and the onset of both depression and CVD, and this association is believed to stem from common pathways, such as inflammation and the activation of the hypothalamic−pituitary−adrenal (HPA) axis (24). However, there is a lack of empirical evidence to substantiate the combined impact of sleep disturbances and depression on heart failure risk (25). Therefore, we posited that sleep disturbances might influence the connection between depression and CVD. The primary objective of this research was to analyses a substantial amount of population data from the NHANES database spanning from 2007 to 2018 to assess the relationships among sleep disturbances, depression, and CVD. Most importantly, we sought to explore the mediating role of sleep disturbance in the association between depression and CVD, aiming to understand the mechanisms through which depression leads to CVD and providing insights for the management of CVD.

## 2 Materials and methods

### 2.1 Study population

The Centers for Disease Control and Prevention (CDC) spearheads an essential research endeavor in the United States called the National Health and Nutrition Examination Survey (NHANES). Its main goal is to thoroughly evaluate the health and nutritional status of the U.S. populace by gathering sample data that genuinely represent the nation’s demographic diversity. These include health, nutritional, and lifestyle data, which are used to track national health trends and related risk factors. The ethical guidelines for data collection were rigorously adhered to, in line with the protocols set forth by the Institutional Review Board of the National Center for Health Statistics (NCHS), as detailed in the latest version of the Helsinki Declaration. Before any health assessments, the study team thoroughly explained the contents of the informed consent forms to participants, ensuring that all participants fully understood and signed those forms before joining the study, thereby safeguarding the ethical integrity of the research and the informed rights of the participants.

**Figure 1:**
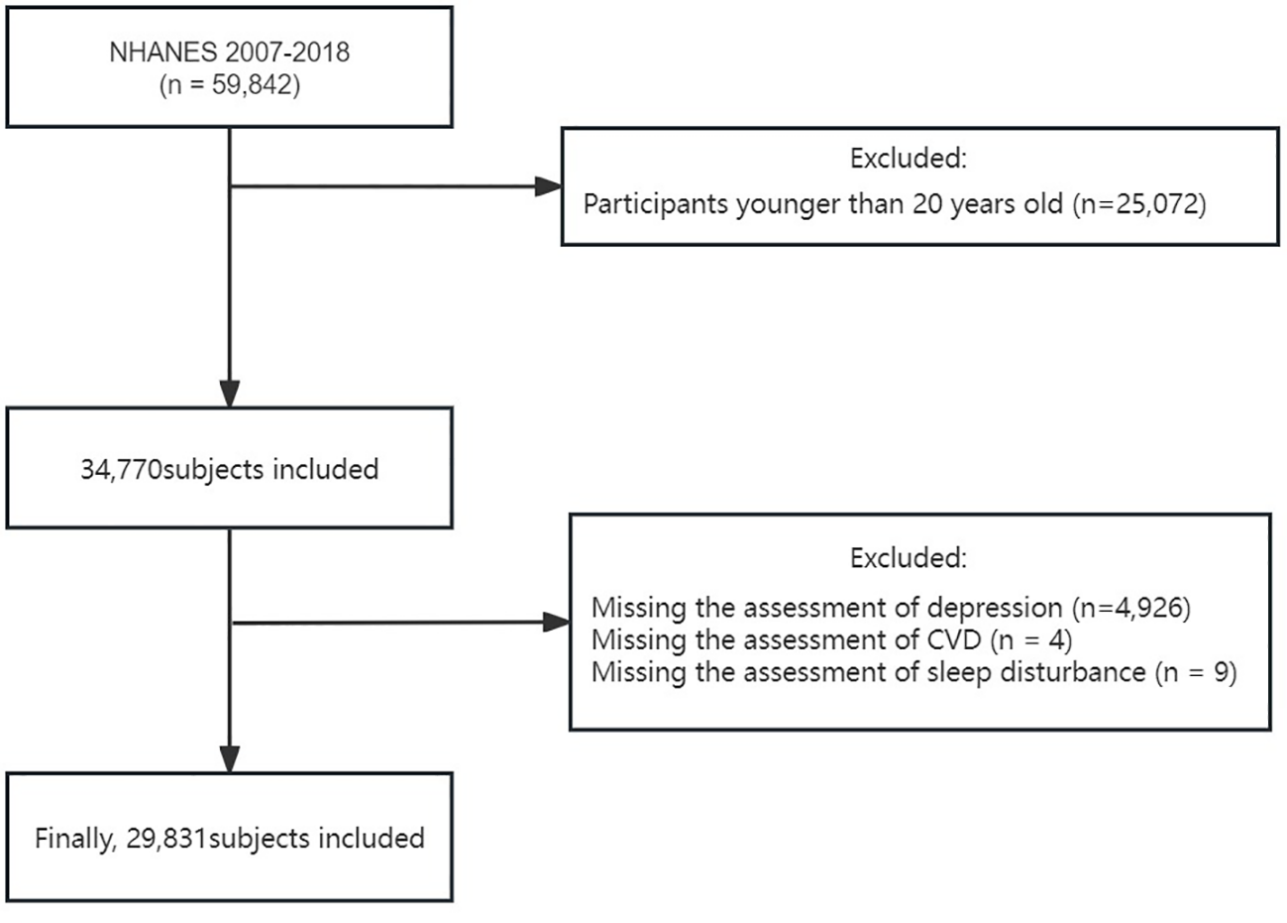
The study’s flow diagram.

In our research, we examined data across six consecutive cycles of the NHANES between 2007 and 2018, encompassing 59,842 participants. Considering that the investigation of the primary study variables was aimed at individuals aged 20 and above, we first excluded participants younger than 20 (25,072 individuals). Subsequently, we further excluded individuals lacking PHQ-9 questionnaire data (4,926 individuals), those without CVD history information (4 individuals), and those lacking sleep disorder information (9 individuals). After these selection criteria were met, the study ultimately included 29,831 participants aged ≥20 years with complete data on depression, sleep disturbance, and CVD (**Figure 1**).

### 2.2 Assessment of primary measures

#### 2.2.1 Assessment of depression

Depression assessment was carried out with the widely accepted Patient Health Questionnaire-9 (PHQ-9) (26), an approved instrument for evaluating symptoms of depression experienced within the previous fortnight. The PHQ-9 was conducted via in-person interviews, with a comprehensive point scale ranging from 0 to 27; elevated scores indicate heightened severity of depressive symptoms. According to the scoring guidelines of the PHQ-9, depression intensity is classified as follows: 0–4 points signifies the absence of depressive symptoms, 5–9 points denotes mild symptoms, 10–14 points suggest a moderate level, 15–19 points indicate moderate to severe symptoms, and 20–27 points indicate severe symptoms. Additionally, attaining a PHQ-9 aggregate score of 10 or above is considered to indicate the presence of depression (27).

#### 2.2.2 Assessment of sleep disturbance

In this study, sleep disturbance was identified based on participants’ self-reported sleep conditions in the NHANES questionnaire. Sleep disturbance was defined as an affirmative response to the question “Have you ever reported sleep problems to a doctor or other health care professional?” This identification method has also been utilized in previous studies (28).

#### 2.2.3 Assessment of CVD

The identification of CVD was based on self-reported diagnoses by doctors and the completion of a standardized questionnaire regarding medical conditions. Participants were questioned about the presence of coronary heart disease, heart attack, congestive heart failure, stroke, or angina pectoris. Those who reported any of these conditions affirmatively were classified as suffering from CVD. Individuals who answered “do not know” or declined to respond were omitted from the analysis.

### 2.3 Covariate assessment

In our study, covariates were carefully selected based on previously described factors related to CVD (26). The covariates included age, sex (female or male), race/ethnicity (Mexican American, Non-Hispanic Black, Non-Hispanic White, or Other), education level (less than high school, high school graduate, college or above), poverty–income ratio (PIR) (<1.3, ≥1.3), marital status (married/cohabiting, divorced/widowed/separated, or single), alcohol consumption status (<12 drinks/year, ≥12 drinks/year), smoking status (<100 cigarettes/lifetime, ≥100 cigarettes/lifetime), body mass index (BMI) (weight (kg)/height (m)^2), self-reported history of hypertension (yes/no), and diabetes status (yes/no/borderline).

### 2.4 Sensitivity analysis

To ensure the stability of the study results, we conducted the following detailed sensitivity analyses. (1) We tested the stability of the mediating effect of sleep disturbance by including specific selective serotonin reuptake inhibitors as covariates. (2) We further investigated the stability of the mediating effect of sleep disturbance by including medications used for treating sleep disturbance as covariates. (3) Given that the risk of depression in women is approximately twice that in men (29) and that research has indicated that there are sex differences in the association between depressive symptoms and CVD (30), we further explored the mediating role of sleep disturbance across both sexes. (4) Finally, considering that late-life depression is a complex, heterogeneous syndrome closely associated with numerous severe health consequences (31), we validated the mediating effects of sleep disturbance after stratification by age (younger than 60 years of age and 60 years and older).

### 2.5 Statistical analyses

The study presented continuous variables as the means ± standard deviations (SDs) and summarized categorical variables as frequencies and percentages. To compare continuous variables among various groups, either the independent samples t test or Mann−Whitney U test was utilized based on the distribution of the variable. For categorical data, differences between groups were assessed using the chi-square test or Fisher’s exact test. We evaluated the associations among depression, sleep disturbance, and CVD using multivariate logistic regression analyses. We constructed three models: Model 1 was an unadjusted/crude model; Model 2 was adjusted for age, sex, and race; and Model 3 was further adjusted for age, sex, race, family PIR, educational level, marital status, alcohol consumption, smoking status, BMI, hypertension history, and diabetes history to control for confounding factors. Finally, we used the bootstrap test method to evaluate whether sleep disturbance plays a mediating role between depression and CVD and to determine the magnitude and statistical significance of that mediating effect.

All the statistical analyses were conducted using R Statistical Software (version 4.3.1, The R Foundation, http://www.R-project.org) and the Free Statistical analysis platform (version 1.9.1, Beijing, China). A two-tailed p value less than 0.05 was considered to indicate statistical significance.

## 3 Results

### 3.1 Baseline characteristics

This study included 29,831 patients, with an average age of 49.9 ± 17.7 years, and males accounted for 50.9% of the population. Among these participants, 10.8% were diagnosed with CVD. As shown in **Table 1**, compared to the non-CVD group, the CVD patient group exhibited distinct characteristics, including a greater proportion of males, older average age, increased percentage of Non-Hispanic White individuals, lower proportion of individuals with higher education attainment, lower average income, more frequent widowed/divorced/separated status, higher rates of smoking and alcohol consumption, higher BMI values, and higher incidence rates of hypertension, diabetes, depression, and sleep disturbance (P<0.05 for all comparisons).

**Table 1:** Baseline characteristics of participants classified by CVD status.

Characteristic	Total (n = 29831)	None-CVD (n = 26583)	CVD (n = 3248)	*P* value
Sex^a^				< 0.001
Male	14658 (49.1)	12804 (48.2)	1854 (57.1)	
Female	15173 (50.9)	13779 (51.8)	1394 (42.9)	
Age^a^, years	49.9 ± 17.7	47.9 ± 17.2	65.9 ± 12.7	< 0.001
Race^a^				< 0.001
Mexican American	4481 (15.0)	4174 (15.7)	307 (9.5)	
Non-Hispanic White	12412 (41.6)	10694 (40.2)	1718 (52.9)	
Non-Hispanic Black	6383 (21.4)	5647 (21.2)	736 (22.7)	
Other race	6555 (22.0)	6068 (22.8)	487 (15)	
Family poverty-income ratio^a^				< 0.001
<1.3	8631 (28.9)	7506 (28.2)	1125 (34.6)	
≥1.3	21200 (71.1)	19077 (71.8)	2123 (65.4)	
Educational level^a^				< 0.001
Below high school	7111 (23.8)	6098 (22.9)	1013 (31.2)	
High-school graduate	6863 (23.0)	6015 (22.6)	848 (26.1)	
College or above	15857 (53.2)	14470 (54.4)	1387 (42.7)	
Marital status, n (%)^a^				< 0.001
Married/living with partner	17650 (59.2)	15893 (59.8)	1757 (54.1)	
Widowed/divorced/separated	6686 (22.4)	5459 (20.5)	1227 (37.8)	
Never married	5495 (18.4)	5231 (19.7)	264 (8.1)	
Alcohol consumption, n (%)^a^				< 0.001
<12 drinks/year	29377 (98.5)	26153 (98.4)	3224 (99.3)	
≥12 drinks/year	454 (1.5)	430 (1.6)	24 (0.7)	
Smoking status, n (%)				< 0.001
<100 cigarettes in life	16513 (55.4)	15260 (57.4)	1253 (38.6)	
≥100 cigarettes in life	13318 (44.6)	11323 (42.6)	1995 (61.4)	
BMI^a^, kg/m^2^	29.3 ± 7.0	29.2 ± 6.9	30.6 ± 7.3	< 0.001
Hypertension, n (%)^a^				< 0.001
No	18856 (63.2)	18040 (67.9)	816 (25.1)	
Yes	10975 (36.8)	8543 (32.1)	2432 (74.9)	
Diabetes, n (%)^a^				< 0.001
No	25082 (84.1)	23093 (86.9)	1989 (61.2)	
Yes	4026 (13.5)	2894 (10.9)	1132 (34.9)	
Borderline	723 (2.4)	596 (2.2)	127 (3.9)	
Depression, n (%)^a^				< 0.001
No	27081 (90.8)	24375 (91.7)	2706 (83.3)	
Yes	2750 (9.2)	2208 (8.3)	542 (16.7)	
SSRI use, n (%)^a^				0.076
No	29302 (98.2)	26099 (98.2)	3203 (98.6)	
Yes	529 (1.8)	484 (1.8)	45 (1.4)	
Sleep disturbance, n (%)^a^				< 0.001
No	22011 (73.8)	20138 (75.8)	1873 (57.7)	
Yes	7820 (26.2)	6445 (24.2)	1375 (42.3)	

CVD, cardiovascular diseases; SSRI, specific selective serotonin reuptake inhibitors. ^a^Continuous variables are presented as the mean ± SD; categorical variables are presented as n (%). ^b^ These characteristics are presented as median (IQR).

### 3.2 Associations between depression, sleep disturbance, and CVD incidence

To delve into the complex interplay between depression, sleep disturbances, and CVD, we conducted a series of studies. As shown in **Table 2**, after multivariate logistic regression analysis and adjustment for potential confounders, patients with depression exhibited a greater risk of CVD than patients without depression did (OR=2.21, 95% CI=1.96 to 2.49; P<0.001). Additionally, the table shows the positive associations between varying degrees of depression and the risk of CVD, with the risk significantly increasing as the severity of depression increased (p value for trend <0.001).

**Table 2:** Association between depression and CVD risk.

Variable	Model 1		Model 2		Model 3	
Depression, n (%)	OR (95% CI)	*P* value	OR (95% CI)	*P* value	OR (95% CI)	*P* value
No	1 (Ref)		1 (Ref)			
Yes	2.21 (2~2.45)	<0.001	3.13 (2.79~3.51)	<0.001	2.21 (1.96~2.49)	<0.001
Severity of depression, n (%)						
None	1(Ref)		1(Ref)			
Mild	1.61 (1.46~1.77)	<0.001	1.93 (1.74~2.14)	<0.001	1.57 (1.41~1.75)	<0.001
Moderate	2.02 (1.77~2.31)	<0.001	2.85 (2.46~3.31)	<0.001	2.02 (1.73~2.37)	<0.001
Moderately severe	2.79 (2.33~3.34)	<0.001	4.29 (3.51~5.23)	<0.001	2.83 (2.3~3.48)	<0.001
Severe	4.18 (3.26~5.37)	<0.001	7.16 (5.43~9.45)	<0.001	4.67 (3.51~6.23)	<0.001
*P* for trend		<0.001		<0.001		<0.001

OR, odds ratio; CI, confidence interval. Model 1 was unadjusted; Model 2 was adjusted for age, sex, and race; Model 3 was adjusted for age, sex, race, family PIR, educational level, marital status, alcohol consumption, smoking status, BMI, hypertension history, and diabetes history.

As shown in **Table 3**, after adjusting for variables such as age, sex, socioeconomic status, and other potential confounders, we identified a markedly elevated risk of sleep disturbance in individuals suffering from depression, as evidenced by an adjusted OR of 4.07 (95% CI=3.73 to 4.44, P<0.001). Additionally, the findings demonstrated a clear positive correlation between the severity of depression and the risk of sleep disturbances, indicating that as depression severity increases, the risk of sleep disturbances significantly increases (p value for trend <0.001). **Table 4** reveals a strong link between sleep disturbances and a greater risk of CVD, even when accounting for multiple risk factors (OR=1.74, 95% CI=1.6 to 1.9; P<0.001). This evidence highlights the critical role that both depression and sleep disturbances play in managing the risk associated with CVD.

**Table 3:** Association between depression and risk of sleep disturbance.

Variable	Model 1		Model 2		Model 3	
Depression, n (%)	OR (95% CI)	*P* value	OR (95% CI)	*P* value	OR (95% CI)	*P* value
No	1(Ref)		1(Ref)			
Yes	4.63 (4.27~5.02)	<0.001	4.68 (4.31~5.08)	<0.001	4.07 (3.73~4.44)	<0.001
Severity of depression, n (%)						
None	1(Ref)		1(Ref)			
Mild	3.05 (2.85~3.26)	<0.001	3.08 (2.88~3.3)	<0.001	2.87 (2.68~3.08)	<0.001
Moderate	5.09 (4.6~5.64)	<0.001	5.23 (4.72~5.8)	<0.001	4.74 (4.25~5.27)	<0.001
Moderately severe	6.61 (5.69~7.68)	<0.001	6.73 (5.78~7.85)	<0.001	5.88 (5.02~6.88)	<0.001
Severe	10.45 (8.15~13.4)	<0.001	10.56 (8.21~13.59)	<0.001	9.12 (7.04~11.8)	<0.001
*P* for trend		<0.001		<0.001		<0.001

OR, odds ratio; CI, confidence interval. Model 1 was unadjusted; Model 2 was adjusted for age, sex, and race; Model 3 was adjusted for age, sex, race, family PIR, educational level, marital status, alcohol consumption, smoking status, BMI, hypertension history, and diabetes history.

**Table 4:** Association between sleep disturbance and CVD risk.

Variable	Model 1		Model 2		Model 3	
Sleep disturbance, n (%)	OR (95% CI)	*P* value	OR (95% CI)	*P* value	OR (95% CI)	*P* value
No	1 (Ref)		1 (Ref)			
Yes	2.29 (2.13~2.47)	<0.001	2.19 (2.02~2.38)	<0.001	1.74 (1.6~1.9)	<0.001

OR, odds ratio; CI, confidence interval. Model 1 was unadjusted; Model 2 was adjusted for age, sex, and race; Model 3 was adjusted for age, sex, race, family PIR, educational level, marital status, alcohol consumption, smoking status, BMI, hypertension history, and diabetes history.

### 3.3 Mediation analysis

**Figure 2:**
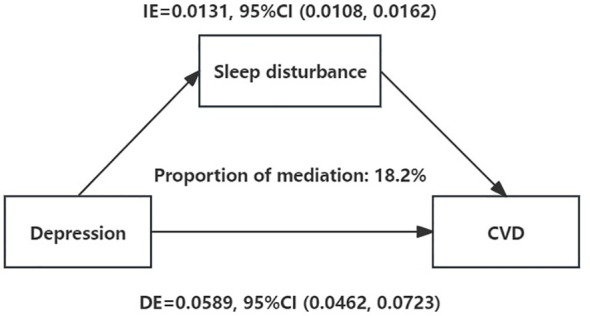
Sleep disturbance as a mediator of the relationship between depression and CVD. Mediation analysis by the bootstrap test: adjusted for age, sex, race, family PIR, educational level, marital status, drinking status, smoking status, BMI, hypertension history, and diabetes history. IE, indirect effect; DE, direct effect; CVD, cardiovascular diseases. *: *P* < 0.05.

To explore the mechanisms by which depression may contribute to CVD risk, a mediation analysis was performed. **Figure 2** shows that after controlling for potential confounders, depression was positively associated with CVD (total effect: β=0.0719, 95% CI=0.0591 to 0.0858, P<0.001). Furthermore, even after adjusting for sleep disturbance risk, the association between depression and CVD risk was still significant (direct effect: β=0.0589, 95% CI=0.0462 to 0.0723, P<0.001). Additionally, the bootstrap test revealed a significant mediating effect (indirect effect: β1 * β2 = 0.013, 95% CI=0.0107 to 0.0161, P<0.001); sleep disturbances mediated the relationship between depression and CVD, contributing to 18.1% of that association.

### 3.4 Sensitivity analysis

Four *post hoc* sensitivity analyses (**Supplementary Figures S1**-**S4**) were conducted to verify the robustness of these associations. The results of the mediation effect remained stable, regardless of whether medications for depression or treatments for sleep disturbance were included as covariates. Additionally, the mediating effect remained unchanged across different age (≥60 or <60 years) and sex subgroups.

## 4 Discussion

This research sought to clarify the complex interrelations among depression, sleep disturbances, and CVD, particularly emphasizing the role of sleep disturbances as a mediator in the association between depression and CVD. Our investigation revealed several key findings: depression and sleep disturbances are significant risk factors for CVD, there is a close relationship between depression and sleep disturbances, and notably, sleep disturbances significantly mediate the relationship between depression and CVD, contributing to 18.1% of the association. This mediating effect was further validated through multiple sensitivity analyses, underscoring its robustness. These insights confirm our initial hypothesis and underscore the critical importance of addressing sleep disturbances in the management of CVD among patients with depression.

Our research indicated that depression is significantly associated with an increased risk of CVD, with the severity of depression being positively associated with an increased risk of CVD. This finding aligns with recent study findings (32). A meta-analysis investigating the prevalence and consequences of CVD in individuals with major depressive disorder identified a significant link between depression and heightened risks of various cardiovascular events (33). The biological link between depression and CVD may be related to dysregulation of the immune system due to overactivation of the HPA (24), along with stress-induced chronic inflammation leading to prolonged high levels of cortisol, which affects vasoconstriction and hypertension, resulting in vascular damage and plaque formation and ultimately leading to CVD (34).

Furthermore, our study revealed that sleep disturbances are significantly linked to a heightened risk of CVD onset. As a standalone risk factor, sleep disturbances are correlated with detrimental cardiac metabolic risks such as obesity, type 2 diabetes, and hypertension (35, 36). Short sleep duration represents one aspect of sleep disturbance. A systematic review of prospective cohort studies indicated a dose−response relationship between short sleep duration and the risk of cardiovascular events (37). Moreover, sleep disturbance includes insomnia, where chronic sufferers often exhibit underlying psychological or physiological characteristics that may promote disease progression and potentially affect CVD risk. Multiple studies have shown that insomnia is related to cardiovascular events (38–42). These findings are in line with our conclusions, underscoring the importance of considering sleep disturbances in the clinical management of CVD risk.

Additionally, our investigation revealed a link between the presence of depression and an elevated risk of experiencing sleep disturbances, with the severity of depression being positively associated with the risk of sleep disturbance. This is consistent with previous research findings (43, 44). Gasperi and Watson et al. proposed that shared genetic and familial factors may partially elucidate the connection between depression and sleep disturbances (45, 46). A twin study revealed that depression is associated with disruptions in rapid eye movement sleep, sleep fragmentation, and variability but not with disruptions in sleep structure or sleep apnoea disorders (47). Sleep disturbances have been identified as an independent diagnostic condition capable of triggering the onset of depression. By improving sleep patterns, the effects of depression can be significantly mitigated (12). Our findings thus contribute to a deeper understanding of the dynamic interplay between depression and sleep disturbances, reinforcing the need for a holistic approach for treating these conditions.

Finally, utilizing mediation analysis for the first time, we investigated the mediating role of sleep disturbance in the relationship between depression and CVD, observing a significant mediating effect, a point that has not been fully affirmed in previous research. Studies by Rao and Dolsen et al. revealed a link between depression and sleep disturbance, suggesting that this connection may be elucidated by the activation of the HPA system and an increase in proinflammatory cytokines (48, 49). Moreover, sleep disturbances can cause dysregulation of the hypothalamic-pituitary axis, alterations in hormones that regulate energy balance and appetite, the initiation of inflammatory responses, molecular impacts on fat cells, insulin resistance, glucose intolerance, changes in gene expression, and vascular calcification through diverse biological pathways (18, 24, 50–52). On the other hand, the activation of inflammatory factors can indirectly affect blood pressure and glucose levels by damaging endothelial cell function and increasing oxidative stress, further exacerbating the risk of CVD (53–55). In summary, depression can lead to sleep disturbance through various mechanisms, thereby influencing the occurrence of CVD events. Finally, it is essential to emphasize that the interaction between sleep disturbance, depression, and CVD is multifaceted, not merely linear or causal. Sleep disturbance could serve as a crucial bridge between depression and CVD, yet the interplay among these conditions is likely shaped by a mix of genetic, environmental, and lifestyle factors. This complex interaction underscores the importance of enhancing sleep quality as a method for the prevention and management of depression and its related cardiovascular issues.

The primary advantage of this research stems from its use of the nationally representative NHANES cohort, signifying an initial effort to delineate the connection between depression, sleep disturbance, and CVD. This finding further verifies that sleep disturbance acts as a partial mediator in the link between depression and CVD. Importantly, the stability of this mediating effect was consistently demonstrated across multiple sensitivity analyses. Additionally, this study is a highly representative national survey covering a broad demographic population, lending significant generalizability to our findings. Despite these strengths, the study also has limitations. Initially, given that the NHANES employs a cross-sectional design, it was not possible to determine the causal relationships among depression, sleep disturbance, and CVD. Subsequent studies should utilize longitudinal cohort studies to elucidate the causal connections between these factors. Additionally, the evaluation of key variables is predominantly based on self-reported data from questionnaires, potentially leading to bias in reporting. Moreover, our study did not account for other potential variables that could affect the outcomes, such as lifestyle factors and genetic predispositions. Finally, as children and adolescents represent another significant demographic of depression but were excluded from this study due to the CVD-related questionnaire targeting only individuals aged 20 and above, caution should be exercised when extrapolating these associations, which were observed in adults, to younger populations.

## 5 Conclusions

Our study illuminates the complex interactions among depression, sleep disturbances, and CVD. By investigating the mediating role of sleep disturbances, we sought to reveal the mechanisms underlying the progression of depression to CVD. Understanding these mechanisms is essential for devising interventions targeting sleep disturbances, thus providing a new approach for managing CVD in patients with depression.

## Data availability statement

Publicly available datasets were analyzed in this study. This data can be found here: (https://wwwn.cdc.gov/nchs/nhanes/Default.aspx).

## Ethics statement

The studies involving humans were approved by The National Center for Health Statistics (NCHS) Ethics Review Board and the Centers for Disease Control and Prevention (CDC) both gave their approval to the survey protocol(Protocol #2011-17, Continuation of Protocol #2011-17), and this study was conducted in accordance with the Declaration of Helsinki (as revised in 2013). All participants gave written informed consent. The studies were conducted in accordance with the local legislation and institutional requirements. The participants provided their written informed consent to participate in this study.

## Author contributions

FC: Conceptualization, Data curation, Formal analysis, Investigation, Methodology, Resources, Software, Supervision, Validation, Visualization, Writing – original draft, Writing – review & editing. HL: Conceptualization, Data curation, Methodology, Software, Writing – original draft, Writing – review & editing. YuaZ: Investigation, Methodology, Software, Supervision, Validation, Visualization, Writing – original draft, Writing – review & editing. YuZ: Conceptualization, Investigation, Supervision, Validation, Visualization, Writing – review & editing. LC: Conceptualization, Investigation, Methodology, Supervision, Validation, Visualization, Writing – review & editing.

## Supplementary material

Supplementary Figure 1 Mediating Effect of sleep disturbance with SSRIs as Covariates. Mediation analysis by the bootstrap test: adjusted for age, sex, race, family PIR, educational level, marital status, drinking status, smoking status, BMI, hypertension history, diabetes history and specific selective serotonin reuptake inhibitors. IE: indirect effect; DE: direct effect; CVD: cardiovascular diseases. *: *P* < 0.05.

Supplementary Figure 2 Mediating Effect of sleep disturbance with sleep disturbance medications as covariates. Mediation analysis by the bootstrap test: adjusted for age, sex, race, family PIR, educational level, marital status, drinking status, smoking status, BMI, hypertension history, diabetes history and sleep disturbance medications. IE: indirect effect; DE: direct effect; CVD: cardiovascular diseases. *: *P* < 0.05.

Supplementary Figure 3 Mediating Effect of sleep disturbance in participants of different genders. A:Male, B:Female. Mediation analysis by the bootstrap test: adjusted for age, race, family PIR, educational level, marital status, drinking status, smoking status, BMI, hypertension history, and diabetes history. IE: indirect effect; DE: direct effect; CVD: cardiovascular diseases. *: *P* < 0.05.

Supplementary Figure 4 Mediating Effect of sleep disturbance in participants of different ages. A:<60, B:≥60. Mediation analysis by the bootstrap test: adjusted for sex, race, family PIR, educational level, marital status, drinking status, smoking status, BMI, hypertension history, and diabetes history. IE: indirect effect; DE: direct effect; CVD: cardiovascular diseases. *: *P* < 0.05.
