# Supplementary figures and images for: The mediating role of sleep disturbance in the relationship between depression and cardiovascular disease
Source: Front Psychiatry. 2024 Jun 5;15:1417179. doi: 10.3389/fpsyt.2024.1417179 (PMC11188478; doi:10.3389/fpsyt.2024.1417179)

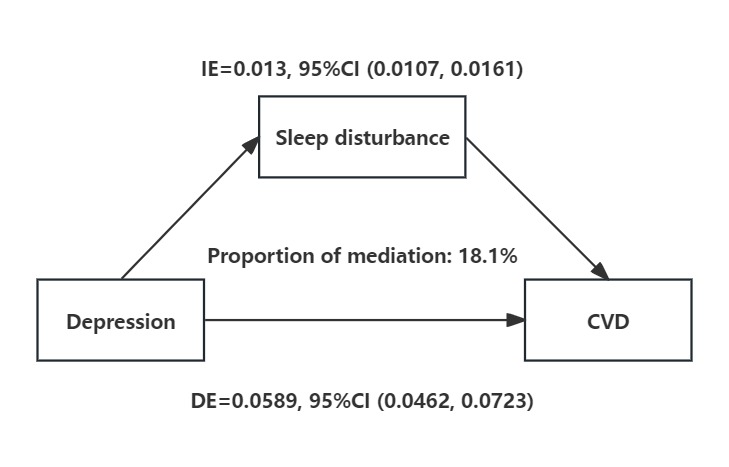

Supplement: Supplementary Figure 1 — Mediating Effect of sleep disturbance with SSRIs as Covariates. Mediation analysis by the bootstrap test: adjusted for age, sex, race, family PIR, educational level, marital status, drinking status, smoking status, BMI, hypertension history, diabetes history and specific selective serotonin reuptake inhibitors. IE: indirect effect; DE: direct effect; CVD: cardiovascular diseases. *: P < 0.05. [file Image_1.jpeg]

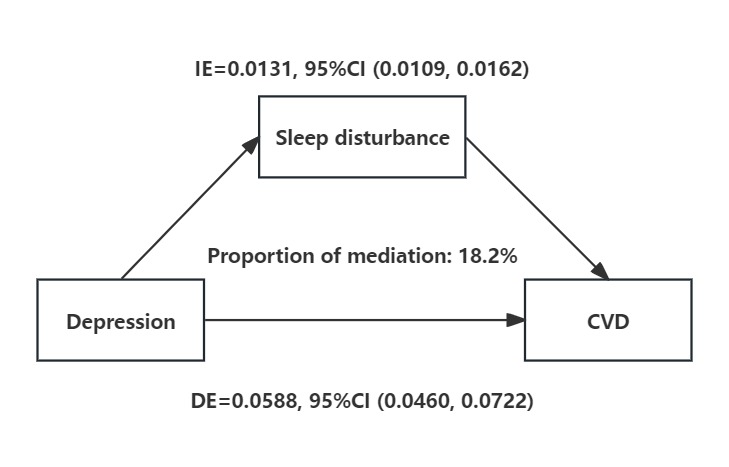

Supplement: Supplementary Figure 2 — Mediating Effect of sleep disturbance with sleep disturbance medications as covariates. Mediation analysis by the bootstrap test: adjusted for age, sex, race, family PIR, educational level, marital status, drinking status, smoking status, BMI, hypertension history, diabetes history and sleep disturbance medications. IE: indirect effect; DE: direct effect; CVD: cardiovascular diseases. *: P < 0.05. [file Image_2.jpeg]

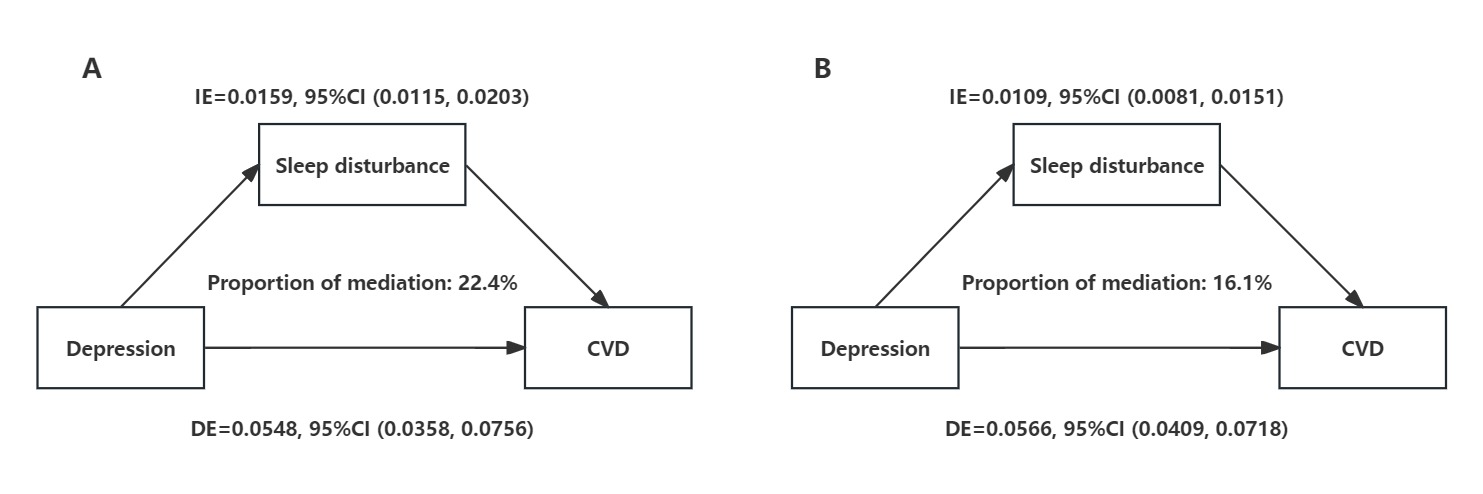

Supplement: Supplementary Figure 3 — Mediating Effect of sleep disturbance in participants of different genders. A:Male, B:Female. Mediation analysis by the bootstrap test: adjusted for age, race, family PIR, educational level, marital status, drinking status, smoking status, BMI, hypertension history, and diabetes history. IE: indirect effect; DE: direct effect; CVD: cardiovascular diseases. *: P < 0.05. [file Image_3.jpeg]

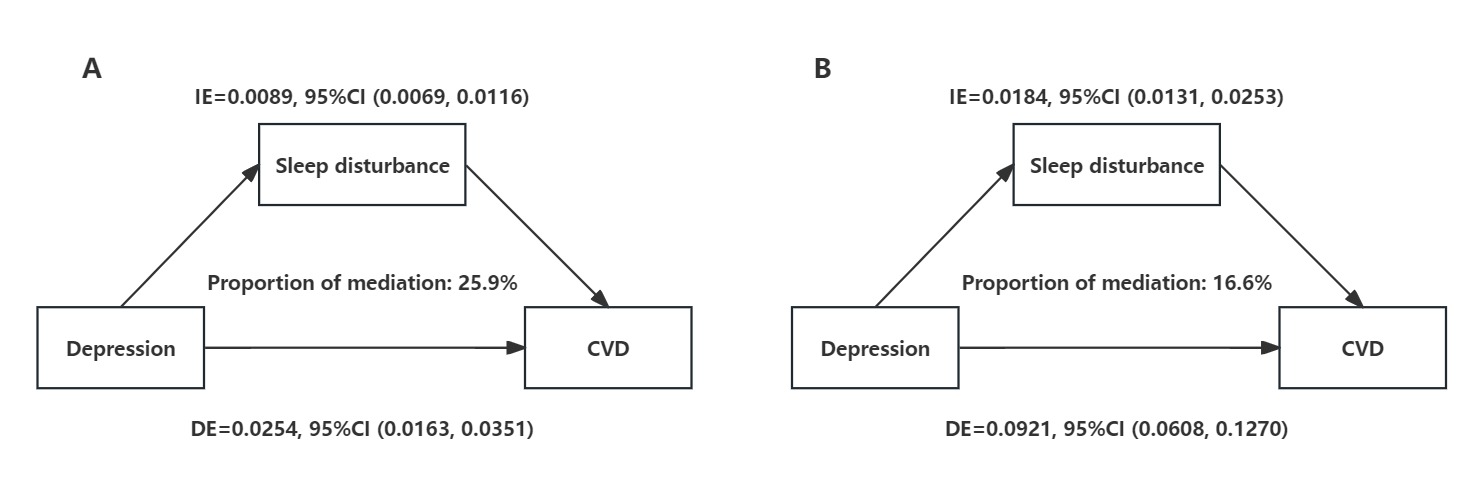

Supplement: Supplementary Figure 4 — Mediating Effect of sleep disturbance in participants of different ages. A:<60, B:≥60. Mediation analysis by the bootstrap test: adjusted for sex, race, family PIR, educational level, marital status, drinking status, smoking status, BMI, hypertension history, and diabetes history. IE: indirect effect; DE: direct effect; CVD: cardiovascular diseases. *: P < 0.05. [file Image_4.jpeg]
